# Supplementary material for: Successful Treatment of Fungal Dermatitis in a Bottlenose Dolphin (Tursiops truncatus)
Source: Microorganisms. 2025 Jan 7;13(1):106. doi: 10.3390/microorganisms13010106 (PMC11767432; doi:10.3390/microorganisms13010106)
Supplement: Supplementary file 1 [file microorganisms-13-00106-s001.zip › 20240102Supplementary_Table_S1.pdf]

Supplementary Table S1. Prescribed antibiotics prior to the antifungal treatment

Antibiotics	Dosage	Day of treatment
Faropenem (876139, Maruho, Osaka, Japan)	4.8 mg/kg P.O. TID [11]	4 to 18
Levofroxacin (876241, Sawai, Osaka, Japan)	5.4mg/kg P.O. SID [11]	18 to 24
Amikacin (876123, FujiPharma, Toyama, Japan)	13.6 mg/kg I.M. SID [11]	24 to 30
Fosfomycin (876135, Meiji Seika Pharma Co., Ltd., Tokyo, Japan)	40.6 mg/kg P.O. BID[*1]	31 to 41
Minocycline (876152, Sawai, Osaka, Japan)	2.0 mg/kg P.O. BID [11]	38 to 47

The initial day the nodule was found on the tail fin of a managed bottlenose dolphin (*Tursiops truncatus*) was designated as day 0. P.O., per os; TID, ter in die; SID, semel in die; I.M., intramuscular injection; and BID, bis in die. Antibiotics doses were followed the previous report in cetaceans [11] and [*1]previous prescription in Port of Nagoya Public Aquarium.
