# Supplementary material for: Successful Treatment of Fungal Dermatitis in a Bottlenose Dolphin (Tursiops truncatus)
Source: Microorganisms. 2025 Jan 7;13(1):106. doi: 10.3390/microorganisms13010106 (PMC11767432; doi:10.3390/microorganisms13010106)
Supplement: Supplementary file 1 [file microorganisms-13-00106-s001.zip › 20250102Supplementary_Table_S2.pdf]

Supplementary Table S2. Prescribed antibiotics during the antifungal treatment

Antibiotics	Dosage	Day of treatment
Levofroxacin (876241, Sawai, Osaka, Japan)	4.8 mg/kg P.O. SID [11]	64 to 124
Minocycline (876152, Sawai, Osaka, Japan)	2.2 mg/kg P.O. BID [11]	104 to 124
Fosfomycin (876135, Meiji Seika Pharma Co., Ltd., Tokyo, Japan)	40 mg/kg P.O. BID[*1]	106 to 116

The initial day the nodule was found on the tail fin of a managed bottlenose dolphin (*Tursiops truncatus*) was designated as day 0. P.O., per os; SID, semel in die; and BID, bis in die. Antibiotics doses were followed the previous report in cetaceans [11] and [*1]previous prescription in Port of Nagoya Public Aquarium.
